# Announcement for 1886

**Published:** 1886-01

**Authors:** 


### ANNOUNCEMENT FOR 1886.

The management of THE JOURNAL feel a just pride in its remarkable success hitherto.

For the ensuing year they have perfected arrangements that cannot fail to make THE JOURNAL more valuable than ever before. Besides the number cf distinguished men all over the country who will continue to write for it, a contract has been made at great expense with Mr. W. K. Tewksbury, a stenographer of national reputation, who becomes the official reporter of THE JOURNAL. He is now engaged in reporting lectures of distinguished men in the colleges in this city, which will appear regularly hereafter ; in addition to which, stenographic reports of the Atlanta Society of Medicine, and of the Medical Association of Georgia, will be supplied. We are determined to make THE AT-

LANTA MEDICAL AND SURGICAL JOURNAL the leading journal of the South, and to this end no expense will be spared.

Our circulation is more than five times as great as it was two years ago, and we will begin with the March issue to publish 5,000 copies—more than double the circulation of any two medical journals in the South.

